# Efficient Gene Targeting in Golden Syrian Hamsters by the CRISPR/Cas9 System

**DOI:** 10.1371/journal.pone.0109755

**Published:** 2014-10-09

**Authors:** Zhiqiang Fan, Wei Li, Sang R. Lee, Qinggang Meng, Bi Shi, Thomas D. Bunch, Kenneth L. White, Il-Keun Kong, Zhongde Wang

**Affiliations:** 1 Department of Animal, Dairy, and Veterinary Sciences, Utah State University, Logan, Utah, United States of America; 2 Department of Animal Science, Division of Applied Life Science, Gyeongsang National University, Jinju, Gyeongnam Province, Republic of Korea; 3 Institute of Agriculture and Life Science, Gyeongsang National University, Jinju, Gyeongnam Province, Republic of Korea; 4 Auratus Bio, LLC., Canton, South Dakota, United States of America; Georgia Regents University, United States of America

## Abstract

The golden Syrian hamster is the model of choice or the only rodent model for studying many human diseases. However, the lack of gene targeting tools in hamsters severely limits their use in biomedical research. Here, we report the first successful application of the CRISPR/Cas9 system to efficiently conduct gene targeting in hamsters. We designed five synthetic single-guide RNAs (sgRNAs)—three for targeting the coding sequences for different functional domains of the hamster STAT2 protein, one for *KCNQ1*, and one for *PPP1R12C*—and demonstrated that the CRISPR/Cas9 system is highly efficient in introducing site-specific mutations in hamster somatic cells. We then developed unique pronuclear (PN) and cytoplasmic injection protocols in hamsters and produced *STAT2* knockout (KO) hamsters by injecting the sgRNA/Cas9, either in the form of plasmid or mRNA, targeting exon 4 of hamster *STAT2*. Among the produced hamsters, 14.3% and 88.9% harbored germline-transmitted *STAT2* mutations from plasmid and mRNA injection, respectively. Notably, 10.4% of the animals produced from mRNA injection were biallelically targeted. This is the first success in conducting site-specific gene targeting in hamsters and can serve as the foundation for developing other genetically engineered hamster models for human disease.

## Introduction

The CRISPR/Cas9 (Clustered Regularly Interspaced Short Palindromic Repeats/CRISPR associated protein 9) system is an RNA-based adaptive immune mechanism to degrade invading plasmids and viruses by bacteria and archaea [1]. It is a nucleoprotein complex composed of a CRISPR coded RNA (crRNA), a trans-activating crRNA (tracrRNA), and a single Cas9 protein. The crRNA, annealed with a tracrRNA, recognizes and directs the Cas9 endonuclease to targeted DNAs in a sequence-specific manner causing their cleavage. It was recently shown that a synthetic sgRNA, by fusing crRNA and tracrRNA, can guide Cas9 endonuclease to target a DNA sequence by design, resulting in site-specific genetic modifications [2], [3]. These landmark studies have led to a string of exciting achievements of highly efficient gene targeting not only in mice but also in several other organisms where homologous recombination-based gene targeting strategy was either not available or extremely inefficient [4]–[12]. However, no success has been reported in employing this system to target the golden Syrian hamster genome.

The golden Syrian hamster (*Mesocricetus auratus*; also referred to as hamster herein) offers unique advantages over other rodent species in modeling certain human diseases. For example, hamsters, but not mice, develop symptoms similar to those observed in humans following infection with hantavirus [13], ebola virus [14], or hendra virus [15]. Among many other human diseases, hamsters are also the preferred model for studying human oral carcinomas [16], pancreatic cancers [17], diet-induced early atheroschlerosis [18], inflammatory myopathies [19], *Clostridium difficile* infection [20], and oncolytic adenoviruses [21]. In addition to disease modeling, hamsters have also been widely used in many other areas of biological research. According to U.S. Department of Agriculture's report on Animals Used in Research, about 146,000 hamsters were used in research in 2010 in the U.S., comprising 13% of total laboratory animal usage (http://www.aphis.usda.gov; accessed 16 September 2013).

However, the inability to target the hamster genome has severely hampered the use of this excellent animal model for biomedical research. Most notably, lack of gene targeting tools has prevented the study of gene pathways and biological processes underlying the pathogenesis of many human diseases for which the hamster is the only suitable rodent model. Here, we report the successful establishment of a CRIPSR/Cas9-mediated gene targeting strategy in the Syrian hamster and the production of hamsters carrying germline-transmitted targeted mutations in the *STAT2* gene. With the Syrian hamster being chosen as one of the 27 high priority eutherian mammals for whole-genome sequencing by the Genome 10K project and the recent completion of draft assembly of its genome (http://www.genome.gov), our study provided a timely technical breakthrough in taping the full potential of this laboratory animal.

## Results

### Design and construction of CRISPR/Cas9 gene targeting vector

**Figure 1:**
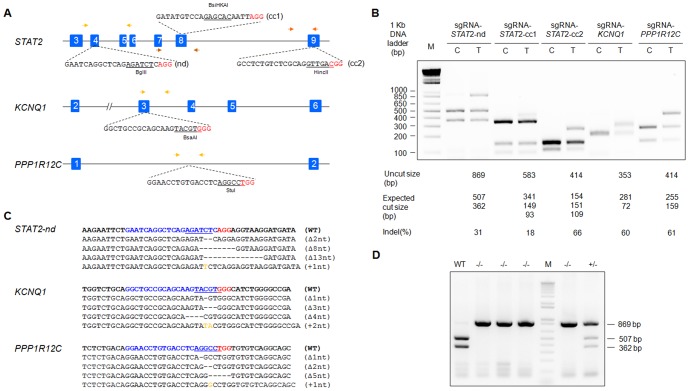
Gene targeting in golden Syrian hamster somatic cells by CRISPR/Cas9. (A) Schematic diagram for the targeting sites at *STAT2*, *KCNQ1* and *PPP1R12C* loci by sgRNAs. The sgRNA target sequences for each locus are depicted, with the restriction enzyme recognition sites used for the PCR-RFLP assays underlined. Letters in red are the protospacer adjacent motif (PAM) (3). Arrows: locations of PCR primers. (B) Gene targeting efficiency at the *STAT2*-nd, *STAT2*-cc1, *STAT2*-cc2, *KCNQ1*, and *PPP1R12C* loci in BHK cells detected by PCR-RFLP assays. M: 1 kb Plus DNA Ladder; C: controls (untransfected BHK cells); T: transfected BHK cells. (C) Indels introduced by CRISPR/Cas9 for the *STAT2*-nd, *KCNQ1*, and *PPP1R12C* loci in BHK cells. Letters in blue are sgRNA target sequences (with PAM in red). Nucleotide deletions and insertions are indicated by dashes and orange letters, respectively. (D) PCR-RFLP assays on single cell derived BHK cell colonies. WT: wild type; Δ1 nt, Δ2 nt, Δ3 nt, Δ4 nt, Δ5 nt, Δ8 nt, and Δ13 nt: one-, two-, three-, four-, five-, eight-, and 13-nucleotide deletion, respectively; +1 nt and +2 nt: one- and two-nucleotide insertions, respectively; -/-: both alleles targeted, and +/-: single allele targeted.

To investigate whether the CRISPR/Cas9 system could be used for gene targeting in golden Syrian hamsters, we designed sgRNA/Cas9 expressing vectors targeting the hamster *STAT2* (one for the N-terminal domain, referred to as sgRNA/Cas9-*STAT2*-nd, and two for the coiled-coil domain, referred to as sgRNA/Cas9-*STAT2*-cc1 and sgRNA/Cas9-*STAT2*–cc2, respectively), *KCNQ1* and *PPP1R12C* genes, respectively (Figure 1A; DNA oligos used for sgRNA/Cas9 vector construction are listed in Table 1). We chose these genes because of their roles in viral infection (*STAT2*) [22], cardiovascular function (*KCNQ1*) [23] or being the most widely used genomic locus for transgene integration (*PPP1R12C*) [24]. To test whether the CRISPR/Cas9 system is effective in introducing targeted mutations in hamster cells, we transfected each of the sgRNA/Cas9 expressing vectors individually into baby hamster kidney (BHK) fibroblasts followed by analyzing the potential gene targeting events by using a PCR-restriction fragment length polymorphism (PCR-RFLP) assay (Figure 1B). We demonstrated that all of the sgRNAs were highly efficient in directing Cas9 to generate targeted cleavages in the hamster genome, with targeting efficiencies being 18%–66% by the three sgRNA/Cas9 expressing vectors for *STAT2*, 60% for *KCNQ1* by sgRNA/Cas9-*KCNQ1*, and 61% for *PPP1R12C* by sgRNA/Cas9-*PPP1R12C* (Figure 1B). To reveal the nature of the genetic mutations introduced by these sgRNA/Cas9 vectors, we focused on analyzing BHK cells transfected by sgRNA/Cas9-*STAT2*-nd, sgRNA/Cas9-*KCNQ1* and sgRNA/Cas9-*PPP1R12C*. We PCR amplified each of the targeting sites, subcloned the genomic PCR products into pCR-blunt vectors, and subjected the resulting plasmids to Sanger sequencing. Our results showed that typical insertions/deletions (indels), caused by repairing double-strand DNA breaks during the error-prone non-homologous end joining (NHEJ) process, were generated in each of the targeted genomic loci (Figure 1C). We also isolated single cell-derived BHK cell colonies by limiting dilution cloning after transfecting the cells with the sgRNA/Cas9-*STAT2*-nd vector. By screening 30 cell colonies with the PCR-RFLP assay, we identified 16 cell colonies as targeted for the *STAT2* gene (16/30; 53.3%), with ten biallelically targeted (10/30; 33.3%) and six monoallelically targeted (6/30; 20.0%; a representative PCR-RFLP assay for these colonies is shown in Figure 1D). Therefore, we demonstrated that CRISPR/Cas9 is a highly efficient system for generating targeted mutations in the golden Syrian hamster genome.

**Table 1:** DNA oligos for sgRNA/Cas9 vector construction and *in vitro* transcription.

Name	Sequence
sgRNA-stat2-ndF	CACCGAATCAGGCTCAGAGATCTC
sgRNA-stat2-ndR	AAACGAGATCTCTGAGCCTGATTC
sgRNA-stat2-cc1F	CACCGATATGTCCAGAGCACAATT
sgRNA-stat2-cc1R	AAACAATTGTGCTCTGGACATATC
sgRNA-stat2-cc2F	CACCGCCTCTGTCTCGCAGGTTGA
sgRNA-stat2-cc2R	AAACTCAACCTGCGAGACAGAGGC
sgRNA-kcnq1F	CACCGGCTGCCGCAGCAAGTACGT
sgRNA-kcnq1R	AAACACGTACTTGCTGCGGCAGCC
sgRNA-ppp1r12cF	CACCGGAACCTGTGACCTCAGGCC
sgRNA- ppp1r12cR	AAACGGCCTGAGGTCACAGGTTCC
Cas9rF	TAATACGACTCACTATAGGGAGATCGCCACCATGGACTATAAGGACCACGAC
Cas9rR	GCGAGCTCTAGGAATTCTTAC
stat2-ndrF	TTAATACGACTCACTATAGGGAATCAGGCTCAGAGATCTC
stat2-ndrR	AAAAGCACCGACTCGGTGCC

### Development of a PN injection protocol in the Syrian hamster

We then focused on developing a PN injection protocol in hamsters for producing genetically targeted animals. In contrast to the situation in other species such as the mouse and rat, Syrian hamster embryos are extremely sensitive to *in vitro* handling. For example, brief exposure of hamster zygotes to light [25] or altering their intracellular pH [26] resulted in a total arrest of embryonic development. Consequently, even though we have extensive expertise in mouse PN injection and routinely perform such procedures in the laboratory, we failed to achieve full-term development from injected hamster embryos using the mouse PN injection protocol. Therefore, we systematically tested various *in vitro* handling conditions to determine which might support full term development. We then identified the best time window post-egg activation (PEA) for injection and optimized the time interval of each round of PN injection, as well as the volume and concentration of sgRNA/Cas9 vector to be injected into a pronucleus. Table 2 summarizes the PN injection conditions we developed for the hamster.

**Table 2:** PN injection conditions in golden Syrian hamsters.

Conditions tested	Conditions identified	Comments
Timing of PN stage embryo collection	10 hours post–egg activation (PEA)	For highest number of PN embryo recovery and most suitable for PN injection
Timing of PN injection	10.5 to 12.5 hours PEA	PN can be best visualized for localization at this time window
Maximum time length embryos can be left on injection stage	60 minutes	15 minutes injection time length is currently used
Maximum DNA injection volume	1–2 pl	>2 pl results in increased embryo lysis rate independent of PN injection time
Maximum DNA concentration	10 ng/µl	>10 ng/µl results in increased embryo lysis rate independent of PN injection time
Embryo handling conditions	Manipulation medium: Equilibrated HECM-9 medium	Equilibrated HECM-9 medium buffered with Hepes may also be used
Ambient environment for embryo handling	The ambient temperature is 28±0.5°C and embryos are handled on a heated plate at 37.5°C	Hamster embryos are sensitive to temperature fluctuations

### Production of *STAT2* KO hamsters by PN injection of DNA

**Figure 2:**
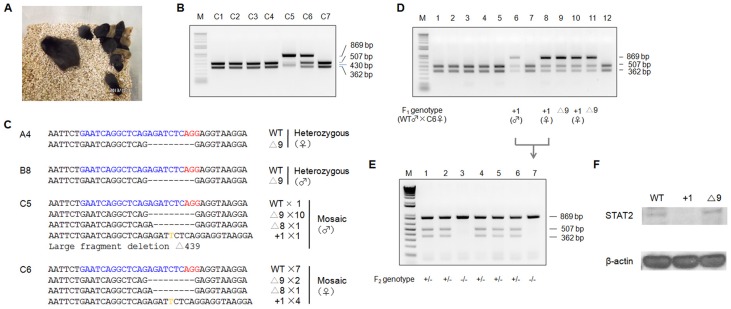
Production of *STAT2*-targeted golden Syrian hamsters by PN injection of sgRNA/Cas9-*STAT2*-nd gene targeting vector. (A) A litter of 2-week-old pups, three golden and seven black, along with the surrogate black mother. (B) Genotyping results by the PCR-RFLP assay on seven golden pups produced by PN injections. Pups C1-C4 and C7 are wild type; pups C5 and C6 are genetically modified in the *STAT2*-nd locus. (C) Indels introduced into the *STAT2*-nd locus of the four golden pups, A4, B8, C5 and C6. The frequencies of DNA subclones with each of the indels are indicated by numbers following the indel signs. (D) Genotyping results with the PCR-RFLP assay on the 12 F1 pups produced by breeding founder animal C6 with a wild type littermate. (E) Genotyping results with the PCR-RFLP assays on the seven F2 pups produced by crossing the two littermates, #6 (male) and #8 (female), both of which are the offspring from founder animal C6. (F) Western blotting assay to compare the expression of STAT2 protein in wild type, homozygous one-nucleotide insertion and nine-nucleotide deletion hamsters. Definitions for symbols are listed in Figure 1 legend.

To investigate whether genetically targeted hamsters can be produced by injecting the CRISPR/Cas9 system using this PN protocol, we chose the sgRNA/Cas9-*STAT2*-nd vector for the test. In total, 215 embryos were injected and transferred into recipients, leading to the birth of 28 golden hamster pups (Table 3). We used black Syrian females mated with black Syrian males the night before embryo transfer (ET) as recipients, because it was reported that *in vitro* manipulated hamster embryos tend to have low viability and are insufficient to signal implantation when transferred to pseudopregnant females [27]. We used hair color to distinguish pups produced from injected embryos from pups produced from natural mating (the black hamster colony is true breeding, i.e. never produced a golden pup without transferring golden embryos into the females). Figure 2A shows a black surrogate mother with her three golden and seven black pups that were at two weeks of age. Genotyping analysis with the PCR-RFLP assays (PCR primer sequences are listed in Table 4) identified that four of the 28 golden pups carry targeted mutations in the *STAT2*-nd locus. Figure 2B shows a representative PCR-RFLP assay by which five golden pups were confirmed as wild type (C1-C4 and C7) and two as genetically modified (C5 and C6). Sanger sequencing analysis of the four targeted golden pups revealed that animal A4 (female) and animal B8 (male) are heterozygous for a nine-nucleotide deletion, animal C5 (male) is genetically mosaic carrying eight-, nine- and 439-nucleotide deletions and one-nucleotide insertion, and C6 (female) is genetically mosaic for eight- and nine-nucleotide deletions and one-nucleotide insertion (Figure 2C). To investigate if the mutations can be transmitted to the germline, we bred all four *STAT2*-targeted hamsters with their wild type littermates. By using the PCR-RFLP assay and Sanger sequencing, we demonstrated that all of the mutations were transmitted to F1 offspring, with the exception of the eight- and 439-nucleotide deletions from the mosaic animals C6 and C5, respectively. Figure 2D shows the genotyping results on the 12 littermates produced by mating the C6 founder female with a wild type male littermate. We have yet to confirm whether the eight- and 439-nucleotide deletions failed to be transmitted to the germline at all or were transmitted with low germline penetrance, since we have only produced the first litters of pups from these two animals so far. We also bred some of the heterozygous F1 littermates and have produced homozygous F2 offspring carrying the nine-nucleotide deletion and the one-nucleotide insertion, respectively (Figure 2E). Western blotting using the tissue lysates from the F2 homozygous hamsters showed that STAT2 protein expression was totally abolished in the one-nucleotide insertion mutants, but not in the nine-nucleotide deletion mutants (Figure 2F; results from the comparison between one STAT2 KO and one nine-nucleotide deletion hamsters are shown). To our knowledge, this is the first successful gene targeting in hamsters and production of live hamsters carrying targeted mutations in their germline. Similar to what has been observed in *STAT2* KO mice [28], *STAT2* targeted hamsters develop and breed normally.

**Table 3:** Production of *STAT2*-targeted golden Syrian hamsters by PN and cytoplasmic injection.

Injection methods	# of transferred embryos	Live Newborns		
		# of golden pups (% of transferred embryos)	# of *STAT2*-modified pups (% of total golden pups)	# of *STAT2*-modified pups (% of transferred embryos)
PN injection (DNA)	215	28 (13.0)	4 (14.3)	4 (1.9)
Cytoplasmic injection (RNA)	229	54 (23.6)*	48 (88.9)**	48 (21.0)**

Note: The data were analyzed by Chi-square test.* *p*<0.01; ** *p*<0.001.

**Table 4:** PCR primers for PCR-RFLP assays.

Category	Name	Sequence	Note
For designated targets	BglF	GTACAGGGAAGAGCTGGAACTGATG	For sgRNA/Cas9-*STAT2*-nd
	BglR	CTGTATCCTCTGTGACACTTGCCAC	For sgRNA/Cas9-*STAT2*-nd
	BsiF	AGAGAAGAAGACACCATCTTTGGAC	For sgRNA/Cas9-*STAT2*-cc1
	BsiR	CTAGCCTCAACTGTCAGTTCTTGTG	For sgRNA/Cas9-*STAT2*-cc1
	HincF	GCCCTGGGTTCTTCTTAGTCAAGTC	For sgRNA/Cas9-*STAT2*-cc2
	HincR	GAGCAGGCGTTGTAGTAACTCTGTG	For sgRNA/Cas9-*STAT2*-cc2
	BsaF	GAGATCGTCCTGGTGGTGTTCTTTG	For sgRNA/Cas9-*KCNQ1*
	BsaR	GCAACTCTTGGTTCTGATGCGGGTC	For sgRNA/Cas9-*KCNQ1*
	StuF	GTCTTTGACCAGTCCAGGAG	For sgRNA/Cas9-*PPP1R12C*
	StuR	TGAATGTCCCTGCTTTCCTG	For sgRNA/Cas9-*PPP1R12C*
For potential off-site targets	O1F	GCACACAGTACAGCAATGGTCG	
	O1R	GCTACACAGAGAAACCCTGACTGG	
	O2F	TTGTGTATAGTGGCAGGTTGGAG	
	O2R	CCGAGTCCAACCGAAACCTATC	
	O3F	CAATGTTCCCTGAGCCTTAAGTG	
	O3R	GTGTATGCATGTCCATGTGTGC	
	O4F	ATGTAACAACCAGGGGTGACTTC	
	O4R	GGCTTCCAACAACTCACACACAC	
	O5F	CTGAGGTAAGGTTTGTGATGTTG	
	O5R	CACACATACACGTCTTCTCCTG	
	O6F	CTCTTGTGGTCTTTGCCAGTTTG	
	O6R	AAGGTCCAAGGAGCCTTGGTGAG	
	O7F	CCAGTTGTTGTCATTTGTGAGAG	
	O7R	ACTCTCCACACCTGCTTCAACTG	
	O8F	GCTAAGAGAATGTGTAAACTCAC	
	O8R	TCTCCAGTCTAAATATCATAGTGG	

### Analysis of off-site targeting in *STAT2* KO hamsters

**Figure 3:**
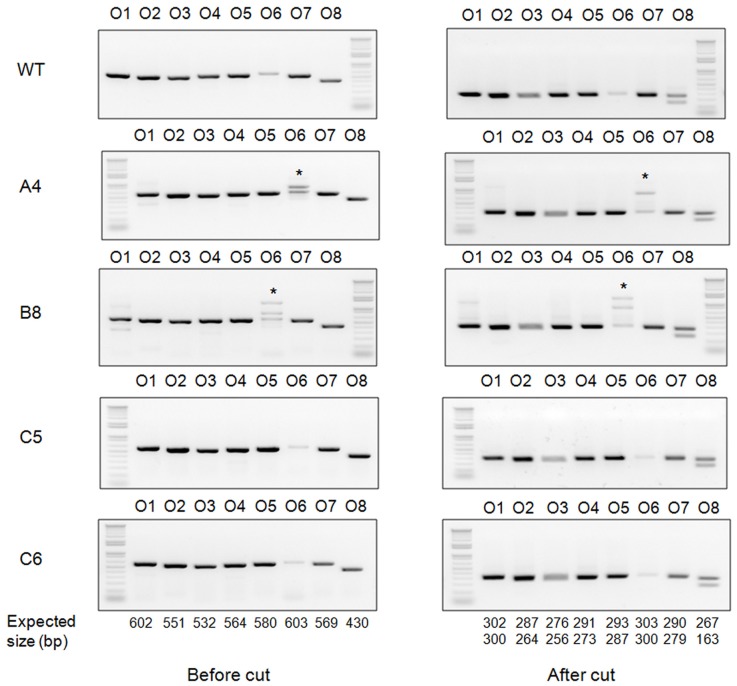
Detection of off-site targeting with PCR-RFLP assays in hamsters produced by DNA injection. Genomic DNA isolated from toes clipped from the four targeted hamsters was used for detecting the off-site targeting with the PCR-RFLP assays. O1-O8: the eight potential off-site targets. Left panel shows PCR products before restriction enzyme digestion and the right panel shows PCR products after restriction enzyme digestion (note the band size changes after restriction enzyme digestions). *: multiple PCR bands due to non-specific DNA amplification (the bottom band is the correct PCR product).

It was reported that CRISPR/Cas9 can introduce off-site targeting [29]. To examine whether off-site targeting occurred in the *STAT2*-targeted hamsters, we conducted blast search on the golden Syrian hamster nucleotide sequence database (http://www.ncbi.nlm.nih.gov/genome/11998) with the targeting sequence by sgRNA/Cas9-*STAT2*-nd as the query to find the genomic sequences with the highest homology, i.e., zero to two nucleotide mismatches in the seed sequence [3]. In total, we chose eight potential off-site targets (O1 to O8) which share the highest sequence homology to the seed sequence of the target site (Table 5) and subjected them to PCR-RFLP assays by using the genomic DNA isolated from *STAT2*-targeted hamsters. Our results showed that none of these sequences was targeted by the sgRNA/Cas9-*STAT2*-nd vector (Figure 3), even though they share high degrees of sequence homology with the targeting sequence. This result indicates that the CRIPSR/Cas9 system is specific in introducing targeted mutations into the golden Syrian hamster genome.

**Table 5:** Top eight potential off-targets by sgRNA/Cas9-*STAT2*-nd.

Sites	Sequences*	Sequence ID and location of sites	Restriction enzymes
O1	ggtTCAGaCTCAGAG*c*TCTC***AGG***	Sequence ID: NW_004801690.1 Range 23: 4636728 to 4636747	SacI
O2	ggccCAGGCTCAGAGA*c* CTC ***AGG***	Sequence ID: NW_004801690.1 Range 1: 4734434 to 4734452	Bsu36I
O3	catggAGGCTCAGAGAT*a*TC ***AGG***	Sequence ID: NW_004801622.1 Range 2: 11824588 to 11824605	EcoRV
O4	gtacagGGCT*g*AGAGATCTC***AGG***	Sequence ID: NW_004801633.1 Range 3: 3439805 to 3439821	BglII
O5	tttctgtGCTCAGAGATCTC***AGG***	Sequence ID: NW_004801707.1 Range 1: 1561837 to 1561852	BglII
O6	tcttgctGCTCAGAGATCTC***AGG***	Sequence ID: NW_004801815.1 Range 1: 759646 to 759661	BglII
O7	ctaggatcCTCAGAGATCTC***AGG***	Sequence ID: NW_004801691.1 Range 1: 1923240 to 1923254	BglII
O8	tgtcacttCTCAGAGATCTC***AGG***	Sequence ID: NW_004801670.1 Range 1: 5134136 to 5134150	BglII

*PAM is indicated in bold and italic letters. Nucleotide mismatches between the target sequence and the potential off-target sequences are in lower-case, with the ones within the seed sequence italicized.

### Production of *STAT2* KO hamsters by cytoplasmic injection of RNA

**Figure 4:**
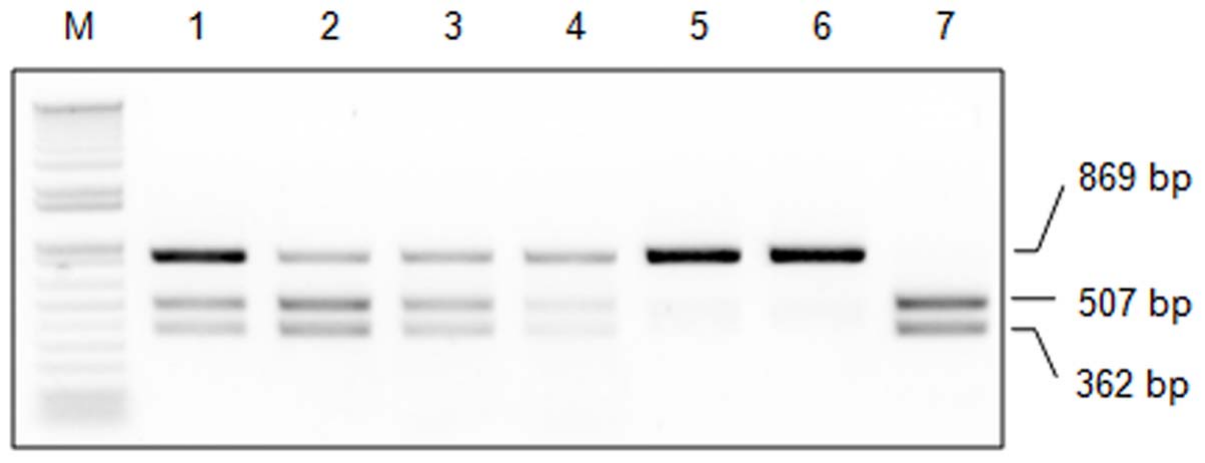
Genotyping with PCR-RFLP assays of *STAT2*-targeted golden Syrian hamsters produced by cytoplasmic injection of *Cas9* mRNA and sgRNA. Typical genotyping results by the PCR-RFLP assay from seven animals are shown. M: 1 kb Plus DNA Ladder; 1–4: heterozygously or mosaically targeted; 5–6: biallelically targeted; 7: wild type.

**Figure 5:**
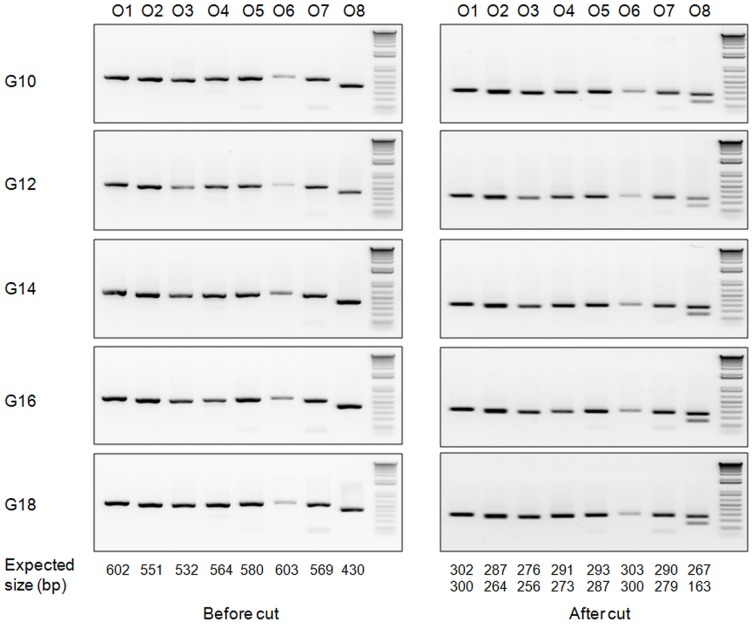
Detection of off-site targeting with the PCR-RFLP assays in hamsters produced by RNA injection. Assay results for five of the 25 analyzed animals are shown. G#: IDs for the analyzed animals. Definitions for symbols and other information are provided in the legend of Figure 3.

Following the success of producing genetically targeted hamsters with this PN injection protocol, we evaluated the feasibility and efficiency of coinjection of *in vitro* transcribed Cas9 mRNA and sgRNA to produce genetically targeted hamsters. After optimizing the conditions for cytoplasmic injection (detailed in Methods section), we coinjected Cas9 mRNA and the sgRNA for *STAT2*-nd into the cytoplasm of PN stage embryos, followed by transferring the injected embryos to recipient females. Our results showed that coinjection of Cas9 mRNA and sgRNA is highly efficient in targeting the hamster genome, with 48 of the 54 (88.9%) golden hamsters produced being genetically modified (Table 3). Figure 4 shows a representative genotyping result for the produced animals with the PCR-RFLP assay. Compared to injecting DNA vectors expressing sgRNA/Cas9, RNA injection is significantly more efficient in introducing targeted mutations in the hamster (*p*<0.001; Table 3). Among the targeted hamsters, five were identified as biallelically targeted and the rest as heterozygously/mosaically targeted (carrying a wild type allele along with one or more types of indels) in the *STAT2* locus by Sanger sequencing. We subjected five of the biallelically targeted and 20 randomly chosen heterozygously/mosaically targeted hamsters to off-targeting analysis and found no off-targeting event (Figure 5). Therefore, we demonstrated that coinjection of Cas9 mRNA and sgRNA is highly efficient and specific at targeting the hamster genome, at a rate very similar to those observed in mice [11] or rats [5]. Furthermore, we found that the percentage of transferred embryos developing to live pups from RNA injection is significantly higher than from DNA injection (Table 3; *p*<0.01). It is likely that cytoplasmic injection is less detrimental than PN injection.

## Discussion

The production of genetically modified laboratory animals has greatly advanced our understanding of gene function and disease processes. Mice are by far the most commonly used mammalian genetic models, largely due to the fact that embryonic stem (ES) cells were first derived in mice and that homologous DNA recombination-mediated gene targeting technology was first established in mouse ES cells [30]. With the recent development of engineered endonucleases, such as transcription activator-like effector nucleases (TALENs) [31] and CRISPR/Cas9 [2], [3], genes have been targeted with high efficiency not only in mice but also in several other model organisms previously considered to be unamenable to gene targeting [8], [9], [32]–[34].

In this study, we first investigated whether the CRISPR/Cas9 system is efficient in introducing targeted mutations in Syrian hamster genome. By constructing five different sgRNA/Cas9 expressing vectors designed to target distinct genomic loci, we demonstrated that the CRISPR/Cas9 system is highly efficient in introducing site-specific genetic mutations in the hamster genome when transfected into BHK cells. We then developed PN and cytoplasmic microinjection protocols for introducing the CRISPR/Cas9 system into PN stage hamster embryos to produce hamsters carrying targeted genetic modifications. We achieved this by systematically testing the *in vitro* embryo handling conditions that are permissive for hamster embryo development and by optimizing the microinjection parameters. We further demonstrated that injection of the CRISPR/Cas9 system into PN stage embryos is highly efficient at producing germline-transmitted site-specific genetic modifications in hamsters. Our work also led to the production of hamsters carrying germline-transmitted targeted mutations in both of the *STAT2* alleles. We also further demonstrated that, by examining each of the hamster genomic loci that shares the highest sequence homology with the targeting sequence in the STAT2 gene, the CRISPR/Cas9 system is highly specific and did not generate any off-targeting event in these examined loci of the produced hamsters. Even though we currently cannot totally rule out the possibility that other yet to be characterized sequences in the hamster genome sharing high homology with the targeting sequence in the STAT2 gene could also have been targeted, our data are in agreement with the recent findings revealed by high-coverage whole-genome sequencing (the most thorough approach employed to date in analyzing off-targeting events) that the incidents of off-targeting by CRIPSR/Cas9 is relatively low [35], [36]. Nevertheless, we are planning to conduct more extensive sequence analysis, such as with whole-genome sequencing, in the STAT2 KO and other genetically engineered hamsters that are being created in our laboratory to provide a thorough assessment on the off-targeting issue.

Considering the fact that the golden Syrian hamster has been used to study several human diseases for which no other rodent models are suitable [13]–[15], the work presented here should complement other model organisms in the study of human disease.

## Materials and Methods

### Gene targeting in BHK cells

The sgRNA/Cas9 gene targeting vectors were constructed by using the pX330-U6-Chimeric_BB-CBh-hSpCas9 plasmid (Addgene ID: 42230) as described by Cong L *et al*. [2]. The sgRNA/Cas9 targeting sites for each of the genomic loci of interest were identified by searching for the G(N)_20_GG motifs. The corresponding DNA oligos (listed in Table 1) for each of the targeting sites were synthesized by Integrated DNA Technologies (Iowa, USA). The final constructs were confirmed by Sanger sequencing. Baby hamster kidney fibroblasts (BHK; ATCC) were cultured in Minimum Essential Medium (MEM) supplemented with 10% fetal bovine serum (FBS), non-essential amino acids, and Penicillin-Streptomycin (Life Technologies). Five µg of circular sgRNA/Cas9 vectors were transfected into 10^6^ BHK cells using Amaxa 4D-Nucleofector (Program No. CA-137; Lonza). Two days post transfection, cells were harvested for genomic DNA isolation by using Puregene Core Kit A (Qiagen) following the manufacturer's protocol. Each of the target genomic loci was PCR amplified from the genomic DNA isolated from BHK cells or hamster biopsies by Phusion High-fidelity DNA polymerase (Thermo Scientific) with the PCR primers listed in Table 4. After digestion with the chosen enzyme, the PCR products were resolved on a 1% agarose gel and stained with SYBR green dye (Life Technologies). Based on whether the PCR products were fully or partially resistant to digestion by a chosen restriction enzyme (indels introduced by sgRNA/Cas9 vectors would abolish the restriction recognition site), indels were detected. To determine gene targeting efficiency by each of the sgRNA/Cas9 vectors, the relative intensities of uncut band and cut bands were analyzed by using the Image J software (1.47p, NIH).

### Animals

Golden Syrian hamsters used for PN embryo production were bred in-house by using founder animals purchased from Charles River (LVG Golden Syrian Hamster, Strain Code: 049). Black Syrian hamsters used as recipients for embryo transfer were from a breeding colony established in our laboratory. All hamsters were raised and maintained in an air-conditioned room with a 14L:10D light cycle (light from 0600).

### Ethics Statement

The experiments were conducted in strict accordance with guidelines of the AAALAC-accredited Laboratory Animal Research Center at the Utah State University and approved by the Institutional Animal Care and Use Committee of Utah State University (IACUC Protocol: 2091). All surgery was performed under by Ketamine/Xylazine anesthesia, and all efforts were made to minimize suffering.

### Embryo Manipulations

Eight to 12 week old female golden Syrian hamsters were induced to superovulate by an i.p. injection of 10-20 IU (indexed to body weight as described by McKiernan and Bavister) [37] of PMSG (Sigma-Aldrich) at 9:00 AM on the day of post-estrus discharge (Day 1 of estrous cycle). Females were mated to fertile males at 7:00 PM on Day 4 of the estrous cycle. PN embryos were collected from oviducts approximately 19 h after mating. Embryos were flushed from oviducts with warmed and equilibrated HECM-9 medium supplemented with 0.5 mg/ml human serum albumin (Sigma-Aldrich). All embryos were then washed twice, transferred into 20 µl drops of HECM-9 covered by mineral oil (Sigma-Aldrich) in groups of 7-10 in a culture dish and cultured at 37.5°C under 10% CO_2_, 5% O_2_, and 85% N_2_. Culture dishes were pre-equilibrated for at least 5 h before use. The experiments were performed in a dark room with a small incandescent lamp, and red filters were used on the microscope light source, as described by Takenaka *et al*. [25].

The microinjection station set up for PN injections in the hamster is the same as the one developed for the mouse [38], but with embryo manipulation conditions uniquely developed for the hamster (for details see Table 1). In brief, fully equilibrated HECM-9 (37.5°C, 10% CO_2_, 5% O_2_, and 85% N_2_) covered by mineral oil was used as injection medium. A group of 7–10 embryos were transferred to a 50 µl HECM-9 drop on the microinjection dish and 1–2 pl of DNA solution was injected into the pronucleus of an embryo; the injection for each group of embryos was finished within 15 min. Microinjections were performed on a heated microinjection stage (37.5°C) with red filters and all the embryo handling procedures were performed in a dark room with a small incandescent lamp (13 W and 120 V). After injection, embryos were washed twice with equilibrated HECM-9 and cultured in HECM-9 covered by mineral oil for at least 0.5 h before embryo transfer. For PN injection, the injection solution was supercoiled DNA purified by using the QIAquick Gel Extraction Kit (Qiagen) and diluted to 2 ng/µl with TE buffer (10 mM Tris-HCl/0.1 mM EDTA, pH 7.5). For cytoplasmic injection, the injection solution was *in vitro* transcribed Cas9 mRNA (100 ng/µl) and sgRNA (50 ng/µl) dissolved in TE buffer and mixed at 1∶1 volume ratio. About 5–10 pl RNA solution was injected into the cytoplasm of an embryo. Injected embryos with normal morphology were transferred to each oviduct (10–20 embryos per oviduct) of recipient black Syrian females which were naturally mated with black males 1 day previously. Recipients were allowed to naturally deliver and raise their pups.

### Western blotting

Ear biopsies were collected from the wild type, homozygous one-nucleotide insertion and nine-nucleotide deletion mutants. Tissues were grinded in liquid nitrogen and then lysed in lysis buffer (150 mM NaCl, 1.0% Triton X-100 and 50 mM Tris, pH 8.0, with protease inhibitor cocktails (Sigma)). Gross protein mass was quantified by Pierce BCA protein assay kit (Thermo Scientific) according to the manufacturer's protocol. Tissue lysates were separated on NuPAGE 4–12% Bis-Tris gel (Life Technologies) and then electrotransferred to PVDF membrane (Life Technologies). The blocking and washing steps were performed using the WesternBreeze chemiluminescent kit (Life Technologies) and strictly followed the manufacturer's protocol. The membrane was incubated with the primary antibody rabbit polyclonal anti-STAT2 antibody (sc-839, Santa Cruz) or mouse polyclonal anti-β-actin antibody (sc-47778, Santa Cruz) for 1 h. After washing, the membrane was incubated with the alkaline phosphatase conjugated secondary antibody (anti-rabbit or anti-mouse) for 30 min and was exposed to X-ray film for 1–5 min.

## References

[1] Garneau JE, Dupuis ME, Villion M, Romero DA, Barrangou R, et al (2010) The CRISPR/Cas bacterial immune system cleaves bacteriophage and plasmid DNA. Nature 468: 67–71. 21048762 10.1038/nature09523

[2] Cong L, Ran FA, Cox D, Lin S, Barretto R, et al (2013) Multiplex genome engineering using CRISPR/Cas systems. Science 339: 819–823. 23287718 10.1126/science.1231143 PMC3795411

[3] Mali P, Yang L, Esvelt KM, Aach J, Guell M, et al (2013) RNA-guided human genome engineering via Cas9. Science 339: 823–826. 23287722 10.1126/science.1232033 PMC3712628

[4] Friedland AE, Tzur YB, Esvelt KM, Colaiacovo MP, Church GM, et al (2013) Heritable genome editing in C. elegans via a CRISPR-Cas9 system. Nat Methods 10: 741–743. 23817069 10.1038/nmeth.2532 PMC3822328

[5] Li D, Qiu Z, Shao Y, Chen Y, Guan Y, et al (2013) Heritable gene targeting in the mouse and rat using a CRISPR-Cas system. Nat Biotechnol 31: 681–683. 23929336 10.1038/nbt.2661

[6] Mao Y, Zhang H, Xu N, Zhang B, Gou F, et al (2013) Application of the CRISPR-Cas system for efficient genome engineering in plants. Mol Plant 6: 2008–2011. 23963532 10.1093/mp/sst121 PMC3916745

[7] Shan Q, Wang Y, Li J, Zhang Y, Chen K, et al (2013) Targeted genome modification of crop plants using a CRISPR-Cas system. Nat Biotechnol 31: 686–688. 23929338 10.1038/nbt.2650

[8] Sung YH, Kim JM, Kim HT, Lee J, Jeon J, et al (2014) Highly efficient gene knockout in mice and zebrafish with RNA-guided endonucleases. Genome Res 24: 125–131. 24253447 10.1101/gr.163394.113 PMC3875853

[9] Tan W, Carlson DF, Lancto CA, Garbe JR, Webster DA, et al (2013) Efficient nonmeiotic allele introgression in livestock using custom endonucleases. Proc Natl Acad Sci U S A 110: 16526–16531. 24014591 10.1073/pnas.1310478110 PMC3799378

[10] Tzur YB, Friedland AE, Nadarajan S, Church GM, Calarco JA, et al (2013) Heritable custom genomic modifications in Caenorhabditis elegans via a CRISPR-Cas9 system. Genetics 195: 1181–1185. 23979579 10.1534/genetics.113.156075 PMC3813848

[11] Wang H, Yang H, Shivalila CS, Dawlaty MM, Cheng AW, et al (2013) One-step generation of mice carrying mutations in multiple genes by CRISPR/Cas-mediated genome engineering. Cell 153: 910–918. 23643243 10.1016/j.cell.2013.04.025 PMC3969854

[12] Yu Z, Ren M, Wang Z, Zhang B, Rong YS, et al (2013) Highly Efficient Genome Modifications Mediated by CRISPR/Cas9 in Drosophila. Genetics 195: 289–291. 23833182 10.1534/genetics.113.153825 PMC3761309

[13] Safronetz D, Ebihara H, Feldmann H, Hooper JW (2012) The Syrian hamster model of hantavirus pulmonary syndrome. Antiviral Res 95: 282–292. 22705798 10.1016/j.antiviral.2012.06.002 PMC3425723

[14] Ebihara H, Zivcec M, Gardner D, Falzarano D, LaCasse R, et al (2013) A Syrian golden hamster model recapitulating ebola hemorrhagic fever. J Infect Dis 207: 306–318. 23045629 10.1093/infdis/jis626 PMC3532827

[15] Guillaume V, Wong KT, Looi RY, Georges-Courbot MC, Barrot L, et al (2009) Acute Hendra virus infection: Analysis of the pathogenesis and passive antibody protection in the hamster model. Virology 387: 459–465. 19328514 10.1016/j.virol.2009.03.001

[16] Vairaktaris E, Spyridonidou S, Papakosta V, Vylliotis A, Lazaris A, et al (2008) The hamster model of sequential oral oncogenesis. Oral Oncol 44: 315–324. 18061531 10.1016/j.oraloncology.2007.08.015

[17] Takahashi M, Hori M, Mutoh M, Wakabayashi K, Nakagama H (2011) Experimental animal models of pancreatic carcinogenesis for prevention studies and their relevance to human disease. Cancers (Basel) 3: 582–602. 24212630 10.3390/cancers3010582 PMC3756378

[18] Jove M, Ayala V, Ramirez-Nunez O, Serrano JC, Cassanye A, et al (2013) Lipidomic and metabolomic analyses reveal potential plasma biomarkers of early atheromatous plaque formation in hamsters. Cardiovasc Res 97: 642–652. 23241314 10.1093/cvr/cvs368

[19] Paciello O, Wojcik S, Gradoni L, Oliva G, Trapani F, et al (2010) Syrian hamster infected with Leishmania infantum: a new experimental model for inflammatory myopathies. Muscle Nerve 41: 355–361. 19813199 10.1002/mus.21502

[20] Best EL, Freeman J, Wilcox MH (2012) Models for the study of Clostridium difficile infection. Gut Microbes 3: 145–167. 22555466 10.4161/gmic.19526 PMC3370947

[21] Wold WS, Toth K (2012) Chapter three—Syrian hamster as an animal model to study oncolytic adenoviruses and to evaluate the efficacy of antiviral compounds. Adv Cancer Res 115: 69–92. 23021242 10.1016/B978-0-12-398342-8.00003-3

[22] Morrison J, Aguirre S, Fernandez-Sesma A (2012) Innate immunity evasion by Dengue virus. Viruses 4: 397–413. 22590678 10.3390/v4030397 PMC3347034

[23] Jespersen T, Grunnet M, Olesen SP (2005) The KCNQ1 potassium channel: from gene to physiological function. Physiology (Bethesda) 20: 408–416. 16287990 10.1152/physiol.00031.2005

[24] DeKelver RC, Choi VM, Moehle EA, Paschon DE, Hockemeyer D, et al (2010) Functional genomics, proteomics, and regulatory DNA analysis in isogenic settings using zinc finger nuclease-driven transgenesis into a safe harbor locus in the human genome. Genome Res 20: 1133–1142. 20508142 10.1101/gr.106773.110 PMC2909576

[25] Takenaka M, Horiuchi T, Yanagimachi R (2007) Effects of light on development of mammalian zygotes. Proc Natl Acad Sci U S A 104: 14289–14293. 17709739 10.1073/pnas.0706687104 PMC1964859

[26] Squirrell JM, Lane M, Bavister BD (2001) Altering intracellular pH disrupts development and cellular organization in preimplantation hamster embryos. Biol Reprod 64: 1845–1854. 11369617 10.1095/biolreprod64.6.1845 PMC5087321

[27] Barnett DK, Bavister BD (1992) Hypotaurine requirement for in vitro development of golden hamster one-cell embryos into morulae and blastocysts, and production of term offspring from in vitro-fertilized ova. Biol Reprod 47: 297–304. 1391335 10.1095/biolreprod47.2.297

[28] Park C, Lecomte MJ, Schindler C (1999) Murine Stat2 is uncharacteristically divergent. Nucleic Acids Res 27: 4191–4199. 10518610 10.1093/nar/27.21.4191 PMC148693

[29] Ran FA, Hsu PD, Lin CY, Gootenberg JS, Konermann S, et al (2013) Double nicking by RNA-guided CRISPR Cas9 for enhanced genome editing specificity. Cell 154: 1380–1389. 23992846 10.1016/j.cell.2013.08.021 PMC3856256

[30] Capecchi MR (2005) Gene targeting in mice: functional analysis of the mammalian genome for the twenty-first century. Nat Rev Genet 6: 507–512. 15931173 10.1038/nrg1619

[31] Li T, Huang S, Zhao X, Wright DA, Carpenter S, et al (2011) Modularly assembled designer TAL effector nucleases for targeted gene knockout and gene replacement in eukaryotes. Nucleic Acids Res 39: 6315–6325. 21459844 10.1093/nar/gkr188 PMC3152341

[32] Blitz IL, Biesinger J, Xie X, Cho KW (2013) Biallelic genome modification in F(0) Xenopus tropicalis embryos using the CRISPR/Cas system. Genesis 51: 827–834. 24123579 10.1002/dvg.22719 PMC4039559

[33] Hai T, Teng F, Guo R, Li W, Zhou Q (2014) One-step generation of knockout pigs by zygote injection of CRISPR/Cas system. Cell Res 24: 372–375. 24481528 10.1038/cr.2014.11 PMC3945887

[34] Xiao A, Wang Z, Hu Y, Wu Y, Luo Z, et al (2013) Chromosomal deletions and inversions mediated by TALENs and CRISPR/Cas in zebrafish. Nucleic Acids Res 41: e141. 23748566 10.1093/nar/gkt464 PMC3737551

[35] Smith C, Gore A, Yan W, Abalde-Atristain L, Li Z, et al (2014) Whole-Genome Sequencing Analysis Reveals High Specificity of CRISPR/Cas9 and TALEN-Based Genome Editing in Human iPSCs. Cell Stem Cell 15: 12–13. 24996165 10.1016/j.stem.2014.06.011 PMC4338993

[36] Veres A, Gosis BS, Ding Q, Collins R, Ragavendran A, et al (2014) Low Incidence of Off-Target Mutations in Individual CRISPR-Cas9 and TALEN Targeted Human Stem Cell Clones Detected by Whole-Genome Sequencing. Cell Stem Cell 15: 27–30. 24996167 10.1016/j.stem.2014.04.020 PMC4082799

[37] McKiernan SH, Bavister BD (2000) Culture of one-cell hamster embryos with water soluble vitamins: pantothenate stimulates blastocyst production. Hum Reprod 15: 157–164. 10611206 10.1093/humrep/15.1.157

[38] Ittner LM, Gotz J (2007) Pronuclear injection for the production of transgenic mice. Nat Protoc 2: 1206–1215. 17546016 10.1038/nprot.2007.145

